# The Guppy Sex Chromosome System and the Sexually Antagonistic Polymorphism Hypothesis for Y Chromosome Recombination Suppression

**DOI:** 10.3390/genes9050264

**Published:** 2018-05-19

**Authors:** Deborah Charlesworth

**Affiliations:** Institute of Evolutionary Biology, School of Biological Sciences, University of Edinburgh, Edinburgh EH9 3FL, UK; Deborah.Charlesworth@ed.ac.uk; Tel.: +44-131-650-5751

**Keywords:** sexual conflict, sexual dimorphism, genetic

## Abstract

Sex chromosomes regularly evolve suppressed recombination, distinguishing them from other chromosomes, and the reason for this has been debated for many years. It is now clear that non-recombining sex-linked regions have arisen in different ways in different organisms. A major hypothesis is that a sex-determining gene arises on a chromosome and that sexually antagonistic (SA) selection (sometimes called intra-locus sexual conflict) acting at a linked gene has led to the evolution of recombination suppression in the region, to reduce the frequency of low fitness recombinant genotypes produced. The sex chromosome system of the guppy (*Poecilia reticulata*) is often cited as supporting this hypothesis because SA selection has been demonstrated to act on male coloration in natural populations of this fish, and probably contributes to maintaining polymorphisms for the genetic factors involved. I review classical genetic and new molecular genetic results from the guppy, and other fish, including approaches for identifying the genome regions carrying sex-determining loci, and suggest that the guppy may exemplify a recently proposed route to sex chromosome evolution.

“a difference between the sexes in recombination frequency has evolved in close association with the evolution of sex chromosomes” [1]

## 1. Introduction

### 1.1. Sex Chromosomes and Sex Determination

A major question concerning sex chromosome evolution is what has led to the lack of recombination between such chromosome pairs, or between sex-linked regions of these chromosomes. A classic paper by Nei [1] long ago presented a model in which selection favours suppressed recombination, based on the idea that “if sex is determined by two or more genes, it is essential for these genes to be inherited together as a unit. This requires inhibition of recombination between the differentiated segments of the X (or Z) and Y (or W) chromosomes. The recombination in the differentiated segments may be inhibited by an inversion or by a gene or genes controlling the biophysical process of recombination”. However, Nei’s model does not offer a reason why sex might be determined by two or more genes. He used the term “sex determination” to include both control of gender, and also control of secondary sexual characters and fertility, writing that “there are so many sex differences in morphology and physiology, that several or many genes are considered to be necessary for complete sex determination”. Today, these two aspects are generally distinguished, although both are likely to be involved in recombination suppression. 

## 2. Evolution of Separate Sexes and Suppressed Recombination between Sex Determining Factors

A first process that may explain non-recombining fully sex-linked regions of genomes is that evolution of a genetic sex determination system from an ancestor that does not have separate sexes probably often involves two or more genes. A genetic sex determination system cannot evolve from an ancestrally hermaphrodite (or monoecious) state by a single mutation: at least two mutations are needed, one generating females and one males [2,3]. 

For example, a system with male heterogamety can arise by a first mutation leading to females, as male-sterility mutations occur commonly in hermaphrodite plants [3]. A second mutation, causing female sterility, can then produce males, and can spread in a population where females are present if it sufficiently increases the male functioning of the males compared with that of the hermaphrodite ancestor. Since female-sterility is very disadvantageous to females, this mutation is necessarily sexually antagonistic. This two-gene model can generate polymorphisms that are maintained at both the “sex-determining” loci, especially if the genes are closely linked [4]. In this model, a two-gene polymorphism plays an essential role in generating selection for closer linkage, as Nei recognized, and has been formally shown to generate selection for even closer linkage [5], potentially producing a SEX locus in which the two component genes are never separated by recombination. Mutations in two (or more) different genes are developmentally plausible [2], and may explain the evidence that many dioecious plants differ by two genes from non-dioecious close relatives [3].

Note, however, that close linkage of sex-determining genes does not necessarily require any evolutionary change in recombination. No selection for suppressed recombination would be required if the sex-determining mutations arose within a genome region where recombination never occurs or is very infrequent, as has been suggested in papaya [6]. Alternatively, the two mutations might occur in a single gene, and therefore be closely linked. Such single gene origination of two sexes seems unlikely, but might be possible [7].

### 2.1. Turnovers Creating Single Gene Sex Determination

“The first step … is the acquisition of a novel sex-determining gene on one member of an autosomal pair, followed by accumulation of alleles conferring an advantage to that sex. Meiotic recombination between the proto-sex chromosomes is suppressed around the heterologous region to preserve the linkage of these sexually antagonistic genes” [8].

In thinking about animal sex chromosomes, most attention has focused on a different stage of their evolution from that just outlined for the initial evolution of two sexes, which may not apply to many animals, in which separate sexes are often old established. Again, selection occurs for closer linkage between two polymorphic genes, but now the two genetic factors that are proposed to select for linkage are a single sex-determining gene and a partially sex-linked sexually antagonistic polymorphism. Single sex-determining genes can arise by a new gene taking over control of sex determination. Several different situations can, in theory, favour invasion of populations by mutant alleles that can determine sex in the presence of an ancestral sex-determining gene (reviewed in [9]). Many models assume that the new sex-determining gene is advantageous to individuals that carry it (e.g., [10]), including the benefit of avoiding producing aneuploid progeny when an inversion has become fixed on one of the sex chromosome pair [11]. However, turnovers can occur without any such fitness advantage. This can occur if a more dominant sex-determining allele appears in a population whose existing sex-determining factor is less dominant, but differs in no other way; genetic drift in a finite population changes the allele frequency, after which it is expected to return to any frequency on a line of neutral equilibria, and, as dominant alleles do this more quickly than recessive ones, their frequency increases faster than for recessive alleles. Dominant alleles are therefore predicted to replace ones with lesser dominance [12]; the opposite change is also possible under genetic drift, but is less probable [13]. Although the effect is weak, this model suggests that “stronger” alleles, with greater control of gender, might replace weaker ones, possibly leading to changes even in taxa whose sex-determining systems evolved recently, and whose sex chromosomes have not undergone any genetic degeneration. 

Examples of turnovers are well documented. For example, the sex-determining loci are found in different genomic locations in different closely related species from various taxonomic groups of fish, including the rice fishes, Oryzias [14,15], and puffer fishes, Takifugu [16]. The sex-determining gene is known in some of these species, and it has been shown that genes with different functions in the sex-determining pathway have become the “master sex-determining gene”, i.e., that the change did not simply involve movement of this gene to a new location. Transitions between XY and ZW systems have also been documented in reptiles and amphibians (reviewed by [17]), and changes in the sex-determining chromosome pair in Diptera [18]. In these cases, it is usually not known whether the same sex-determining genes are involved, or whether takeovers have occurred—most likely both kinds of events are possible.

It is clearly not inevitable that new non-recombining regions will evolve after a takeover, as a two-gene polymorphism is necessary. One turnover model that does generate this situation, and creates selection for closer linkage, assumes that a sexually antagonistic (SA) polymorphism is maintained at an autosomal locus, such that individuals would benefit if it became linked to the sex-determining gene [10]. For example, a mutation in a gene involved in control of a secondary sexual character in one sex, or a trait under sexual selection, might harm the other sex. If a SA polymorphism is established, it might trigger such a turnover event. SA effects are plausible [19], including in animals in which sexual selection occurs, or similar differences in investment in attractiveness to pollinators by male plants versus females [20]. Levels of expression of many genes may differ in the two sexes of animals [21] and plants [22,23,24], supporting the idea that the two sexes have different evolutionary optima for expression. However, SA polymorphisms establish only under restricted conditions [25], and empirical tests are needed to find out whether such polymorphisms indeed occur and are maintained long enough to cause the effects proposed in theoretical models.

### 2.2. Control of Secondary Sexual Characters: The Sexually Antagonistic Selection Hypothesis

The X and Y chromosome are thought to have evolved from an ordinary pair of autosomes that stopped recombining with each other after acquiring a sex-determining role. The accumulation of sexually antagonistic genes (i.e., genes that are beneficial in one sex but detrimental in the other) linked to the sex-determining genes favours the evolution of suppression of recombination between the nascent sex chromosomes [26].

The trade-offs involved in the kinds of intra-locus sexual conflicts just outlined are especially likely to generate balancing selection and maintain polymorphisms [27] in regions closely linked to an existing sex-determining locus (or a non-recombining region that includes such a gene or genes) [28]. A genome region that has acquired a SEX locus for any reason may therefore experience selection for reduced recombination. 

However, it is difficult to exclude the alternative possibility that suppressed recombination near SEX loci evolved for some other (unknown) reason. If the SA polymorphism hypothesis is correct, and genetic variation for recombination rates exists, populations will often evolve suppressed recombination between the sex-determining, or SEX, locus and the partially sex-linked gene with the SA polymorphism. Once recombination has become suppressed, the region in question will be present as a polymorphism with two haplotypes, for example X- and Y-linked haplotypes, with one haplotype found only in males and carrying the male-determining factor and the male-benefit allele at the sexually antagonistic gene. This situation might be empirically detectable, for example if an inversion has suppressed recombination. However, we will not know that the inversion actually carries a male-benefit allele at the sexually antagonistic gene, because the lack of recombination prevents use of classical genetic methods to test the effects of genes within the region. Moreover, if a non-recombining region has evolved, SA variants are subsequently expected to accumulate within it. If a Y-linked region has not degenerated and lost genes, male-benefit mutations at loci in the region can spread, as they are confined to males and cause no conflict between the sexes. This adds to the difficulty of testing whether they caused selection for the close linkage. 

Furthermore, conflicts can also be resolved by changes in the expression of genes and the phenotypes they produce, and if this occurs in a given conflict situation between the sexes, the recombination rate might remain unchanged. Such a change would again be difficult to detect, because the change producing sex-specific expression would be expected to become fixed in the population [2], making genetic detection impossible after the fixation occurred. Evidence for SA selection therefore comes mainly from genomic analyses, for example tests of the prediction that X chromosomes should accumulate alleles benefitting males, and (by extension) genes with higher expression in males than females [29]. Such accumulation represents fixations of variants, not polymorphisms. Overall, therefore, the SA polymorphism hypothesis is very difficult to test. Its status as a widely accepted hypothesis is due partly to the absence of an alternative. Recently, the generality of this mechanism of sex chromosome evolution has started to be questioned (e.g., [30,31]). The main evidence that supports the hypothesis is that some form of selection has often led to recombination suppression is that recombination suppression has repeatedly evolved between sex-determining loci and genome regions that were formerly partially sex-linked. I therefore next outline the evidence for this trend, which argues against purely neutral processes being involved (although occasional recombination suppression events, such as the spread of a specific inversion, could, of course, have occurred under genetic drift in small populations).

### 2.3. Evolutionary Strata and Sexually Antagonistic Selection

**Figure 1:**
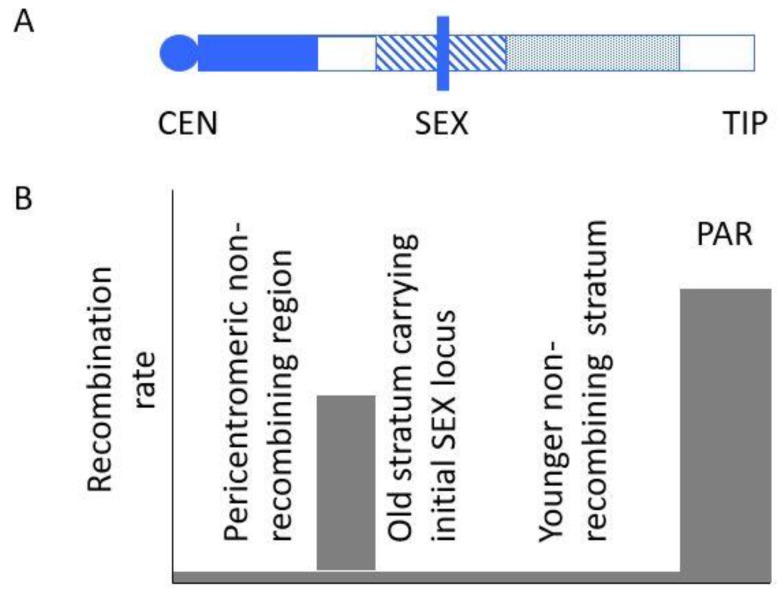
Schematic of patterns of recombination in sex chromosomes. (**A**) Regions with different recombination rates in a hypothetical acrocentric Y chromosome, or a chromosome arm. White indicates regions where recombination occurs, and blue indicates regions with no recombination, with different fill patterns indicating two strata that evolved at different times. (**B**) The grey areas diagram recombination rates in the different regions. Recombination is infrequent, or absent, near the centromere, and also in the two evolutionary strata that have evolved through recombination becoming suppressed in two separate events during the evolution of the sex chromosome pair. The older stratum (diagonal stripes) includes the sex-determining locus (indicated by a vertical bar labelled SEX), and a younger one that subsequently evolved in a more distal region. Both these strata then became completely sex linked. The region proximal to the older stratum might, however, continue to recombine, as illustrated in the figure. If, however, the SEX locus arose within a non-recombining pericentromeric region (or prevented recombination occurring with such a region), the pericentromeric region would also become fully sex linked, and the Y–X divergence in the left-hand region might be as high as in the older stratum. The threespine stickleback XY pair appears to be similar to this situation, with two strata with very different X–Y sequence divergence [37].

Sex chromosomes of several organisms display “evolutionary strata”, defined as contiguous regions that stopped recombining in discrete events (as illustrated in Figure 1). Different events are detectable, because each of them allows sequences to start diverging; for example, in XY systems, Y-linked sequences start to diverge from their ancestral states, as new variants appeared by mutation and became fixed in the population of Y-linked haplotypes (reviewed in [32]). In humans, five such strata are recognised based on estimates of sequence divergence for those infrequent X-linked genes whose alleles are still present in the Y-linked region; divergence was inferred to have started at different times in mammalian evolution, with long intervals between different events [33,34,35]. Independent, successive sex chromosome recombination suppression events are also inferred in birds [36], threespine sticklebacks [37], and some species in the plant genus *Silene* [38]. A case where strata may have formed recently in a fish species was inferred in *Nothobranchius furzeri* [39].

The proximate mechanisms suppressing recombination between sex chromosomes are not currently well understood. In mammals, inversions were probably responsible for at least some of the changes [40]. Small shifts in the boundary between the fully sex-linked and the partially sex-linked, or pseudo-autosomal (PAR), regions have also repeatedly occurred in mammals [41,42], and it is less clear what chromosomal mechanism led to these changes [43]. 

The model outlined for the initial evolution of separate sexes can account for a single non-recombining region, but does not predict multiple strata, such as the five detected in humans. The observation that strata have evolved in the sex chromosomes of a wide range of organisms therefore offers some support for the hypothesis that recombination suppression between sex-determining loci and other genome regions on the same chromosome because of co-adaptation, most likely because SA mutations arose in partially sex-linked genes, became polymorphic, and generated selection for closer linkage. Some form of selective (non-neutral) process was probably involved, since evolution of each new stratum involves shrinkage in the partially sex-linked region, which seems likely often to be deleterious, because it may weaken pairing and lead to univalent production and thus to aneuploid progeny [11]. 

## 3. The Guppy System

The guppy has long been cited as supporting the SA polymorphism hypothesis for recombination suppression between sex-determining and other partially sex-linked genes. Populations of this fish exhibit sexually antagonistic male coloration polymorphisms. Coloration patterns benefit males during mating, but are otherwise harmful to both sexes, as these traits increase predation rates [44,45]. The polymorphisms are not maintained solely by SA selection, but their maintenance is probably aided by being combined with advantages to rare male phenotypes during mating and under predation [46,47]. Guppies may carry other SA polymorphisms, as fish from low predation up-river sites also differ in many ways from those in downstream sites of the same rivers, where predation is intense (reviewed by [48]).

The guppy therefore seems ideal for testing whether SA polymorphisms indeed create selection for sex linkage. Given the difficulty (explained above) of excluding the possibility that evolutionary strata on sex chromosomes evolved for some reason other than the presence of a partially sex-linked SA polymorphism, species in which single sex-determining loci have evolved in recombining genome regions by turnover events are of great interest for studying recombination suppression. The guppy may represent such a species. 

It was established long ago that guppy male coloration traits often exhibit Y-linkage [49], and later work has confirmed that this fish has an XY system [50], albeit one in which the Y-linked region can carry alleles of X-linked genes, unlike the situation in mammals and *Drosophila*, whose Y chromosomes have lost most genes present on the X (reviewed by [51]). Most of the genetic factors controlling the male coloration traits in natural guppy populations are concentrated on the sex chromosomes. Fewer than 20% of these factors are autosomal [52], although the autosomes represent around 96% of the genes, since there are 22 autosomes in a karyotype with roughly similarly sized chromosomes; the estimate that 96% of the genome is autosomal is confirmed by the recent assembly of a female guppy genome sequence [53]. 

Roughly half of the sex-linked factors are fully sex-linked, while the others have been shown to recombine with the sex-determining locus, albeit at rates less than 10% [44,52]. The partially sex-linked factors also show male-limited expression, but partial sex linkage can be detected by treating females with testosterone, causing them to develop as males and express any male coloration factors they carry [44]. The guppy male coloration polymorphisms therefore behave just as predicted for sexually antagonistic mutations, which are expected to establish polymorphisms most readily if closely linked to sex-determining regions, and experience selection for sex-specific expression so long as they remain polymorphic and continue to generate conflicts between the sexes. There is thus little doubt that the guppy sex chromosome carries partially sex-linked polymorphic SA factors.

The SA polymorphism hypothesis predicts that recombination should be less frequent when predation pressure is strong, specifically in down-river sites, as the up-river sites lie above waterfalls that prevent migration of the most important predator (reviewed by [48]). Therefore, females that inherit and express male-benefit traits, such as male coloration, will suffer the greatest loss of fitness in down-river sites. Genetic studies of male coloration indeed indicate genetic differences in linkage in the predicted direction. The only direct evidence comes from a large set of crosses between males from the Aripo river in Trinidad [44]. Thirty-three down-river males were tested, and the *Sb* factor was (controlling a male coloration trait, a reflecting blue spot near the dorsal fin of male fish) transmitted nearly exclusively to their male progeny; co-segregation with the Y-linked region was almost complete, with only one recombinant among the 459 female progeny (a rate of just under 0.1%, see Table 1). In marked contrast, among only 19 up-river males tested (which yielded 387 progeny), several recombinant progeny were observed (Table 2). Although the recombination rate is still low (1.29%), it is about 13 times higher than for down-river males, a significant difference (*p* = 0.007 by a Fisher’s Exact test). Moreover, the *Sb* factor was carried equally often on the up-river parental males’ X and Y chromosomes, and eight of these males were homozygotes, clearly indicating that *Sb* has recombined with the sex-linked region in this population.

It appears that no other trait has been studied in the same way as the *Sb* factor, probably because genetic studies are laborious in the guppy, a schooling fish that does not lay eggs, but bears live progeny, often several weeks after mating. However, experiments in which females sampled from various Trinidad populations were treated with testosterone yield results consistent with differences in recombination rates in the predicted direction. Females from populations in which a coloration factor is fully Y-linked should never show the trait. Female carriers of partially sex-linked traits can, however, express them under testosterone treatment. Indeed, treated females from up-river populations express some male coloration traits more often than ones from down-river sites, consistent with the direct evidence for more frequent recombination in the former [44,54]. Evidence for rapid changes in the frequency with which females carry coloration factors has also been obtained by such experiments, using recently founded populations that have experienced lower predation than the populations from which the founders were sampled [55]. However, these indirect experiments do not distinguish how much of the effect is due to increased recombination, and how much to higher frequencies of coloration alleles. 

Now that molecular markers can be developed for non-model organisms such as the guppy, and, using a genome assembly, for any desired genome region [56,57], it should be possible to test whether the genetic maps differ between up- and down-river populations of guppies. A major advantage of molecular markers in the guppy system is that they can be genotyped in both sexes without the need for hormone treatments. Guppy sex-linked markers have been mapped in crosses between fish from two distinct source populations [58,59], and some of them have been used in fluorescent in situ hybridization (FISH) experiments to identify the sex chromosome, which, uniquely, displays a male-specific heterochromatic region in guppies from various populations [60,61], although the morphology of chromosome 12 of fish from different populations is somewhat variable (FISH, e.g., [60,61,62]). These results suggest that the sex-determining locus is always located on the guppy LG12, even in *P. wingei*, which could be a distinct species [48,63]. In the closely related platyfish, *Xiphophorus maculatus*, the sex-determining factor is on a different chromosome, 21 [64].

### 3.1. Recombination Patterns in Fish

**Figure 2:**
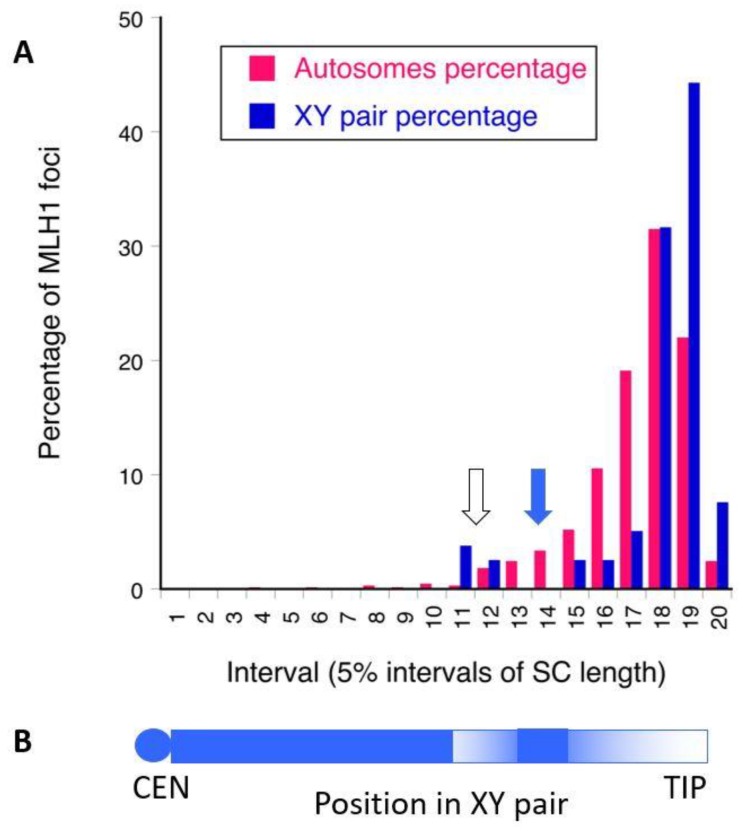
Patterns of recombination in guppy sex chromosomes. (**A**) Crossing over in males. Cytologically estimates proportions of crossover events detected in the autosomes (pink bars) and XY chromosome pair, LG12 (blue bars). All chromosomes are acrocentric. Crossovers were detected by staining for MLH1 protein foci [62], and the numbers shown in that paper were converted to percentages, in order to compare the proportions in different portions of the synaptonemal complex lengths (SC length). Low crossing over across most of the chromosomes in the males studied is not specific to LG12. The arrows indicate a non-recombining region in the distal part of the XY pair (blue arrow), and a more proximal recombining region (white arrow). (**B**) A diagram of the likely recombination pattern in male guppies shows the morphology of the acrocentric sex chromosome pair, with the most highly recombining regions shown as white, and regions with lower frequencies of recombination coloured blue, where different intensity of the blue indicates graded transitions between these region, rather than the sharp boundaries between regions with different recombination rates inferred in organisms with “evolutionary strata”. CEN indicates the centromere of the chromosome and TIP indicates its tip.

A cytogenetic study of guppy males from a non-natural population source found chiasmata localized in the terminal 25% of the XY pair (Figure 2), with few events distal to the terminal 15% of the XY pair [62]. As its total size is about 26.5 Mb [53], this suggests that recombination is mostly restricted to approximately 4 Mb at the tip of the chromosome. A part of the terminal 25% region was apparently non-recombining in the fish examined, and is a candidate for the location of the sex-determining locus [62]; its position corresponds with a region that stains with a male-specific sequence, and it also includes a male-specific heterochromatic region [61]. Rare crossovers were also detected in a small region proximal to this region (Figure 2). This should be tested by genetic mapping, but if recombination is rare large families will be needed. Figure 2 diagrams the recombination pattern expected based on these cytological observations. A pattern of recombination mainly in distal regions in male meiosis, contrasting with events throughout the X in females, is consistent with the available information from high-density mapping of molecular markers in crosses of guppies from natural populations [58,59]; 12/13 crossover locations in male meiosis were distal to 20 cM in the merged map for both sexes, versus none of the 15 events mapped in female meioses.

Recombination may thus be concentrated at the distal chromosome ends in guppy male meiosis, and this is not specific to the XY pair. Pairing in the proximal regions of the XY pair in males [62] might allow gene conversion, which is known to occur in regions that do not undergo crossing over in a range of organisms [65], and, at least in *Drosophila*, conversion events are not suppressed by the “centromere effect” [66]. In the future, tests for gene conversion should also be done. More cytogenetic data, as well as genetic map comparisons between the autosomes and sex chromosomes, are needed. 

**Figure 3:**
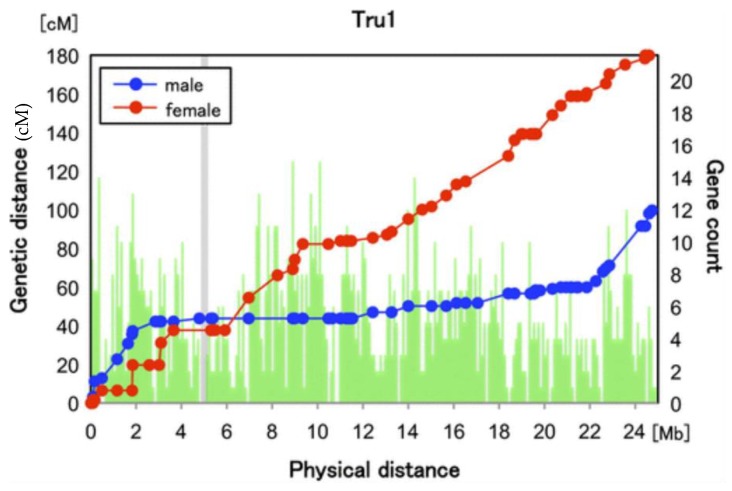
A metacentric fugu chromosome, chromosome 1, showing the different recombination patterns in male and female meiosis, with male recombination events concentrated at the tips of the two chromosome arms. The grey region forming a gap in the vertical green bars (which give the numbers of genes in each 10 Kb bin), indicates a region of the assembly in which centromeric repeats were found, suggesting that this is the centromere of this metacentric chromosome. The map is based on 62 individuals from a full-sib family. Reproduced, with permission, from [73] published by Oxford University Press on behalf of the Society for Molecular Biology and Evolution.

Overall, it is probable that the recombination pattern in guppies is similar to that in other fish. Fish species often have sexually dimorphic recombination rates, with rates generally lower in male than female meiosis, although males are not achiasmate [67]; unlike most *Drosophila* species, some crossing over does occur in males. A few species’ genetic maps are sufficiently detailed to show that crossover events are more localised at the tips of chromosomes in male than female meiosis [67,68,69,70,71], as illustrated in Figure 3 with the example of fugu. The mechanism of this sex difference in crossover locations is unclear, apart from in recently tetraploidised species [72].

If recombination is very rare in males across most of the guppy sex chromosome, a lack of recombination could thus potentially simply be the ancestral state, unconnected with the fact that the chromosome carries the sex-determining factor, as suggested for frog species [74], rather than lower recombination rates having evolved specifically on this chromosome pair. However, this should be tested further, by estimating separate dense genetic maps in male and female guppies. Different natural populations could well differ in their recombination patterns, and chiasma localization at the tip might be stronger for the chromosome 12 pair than for the autosomes. The observation that, in male guppies, univalents were generally seen only for the XY pair [75] suggests that this pair may indeed form a chiasma less often than the autosomes. However, the frequencies of univalents have not been accurately estimated, and it is currently unclear whether there is a statistically significant difference. 

If closer linkage proves to have evolved in some guppy populations through different degrees of localisation of chiasmata in terminal recombining regions of the XY pair, guppies may have no distinct PAR boundary, but simply a region in which the probability of a crossover event in males changes between high and low values (Figure 2B). Inversions and strata are thus not necessarily expected. Testing for strata (or their absence) requires estimating divergence per nucleotide site between Y- and X-linked sequences, preferably for silent or synonymous sites. Such estimates are not yet available from guppy populations, but I next review the currently available relevant information concerning the guppy XY pair from population genomic studies. 

### 3.2. Population Genomic Analysis of the Guppy XY Pair

The guppy sex-determining region has been located to the distal part of the XY pair based on the cytogenetic studies outlined above, and this can now be tested further with modern approaches. In the presence of recombination, fully sex-linked regions can potentially be located by population genomic studies of molecular variants in natural populations: if paired XY regions, such as those proximal to the proposed distally located sex-determining region in guppies, recombine in males, even rarely, this will eliminate associations of variants in the proximal regions with the sexes of fish. Recombination occurring over many generations, even at low rates, allows variants to “migrate” between the X- and Y-linked regions, eventually eliminating associations, and leaving the sex-determining region as a single peak in the proportion of variants showing linkage disequilibrium (LD) with sex. The X and Y haplotypes in a sex-linked region should behave like two sub-populations of the same species, and allele frequency differences between them can potentially be detected by analyses similar to differences between partially isolated populations, such as analysis of *F*_ST_ values (the proportion of sequence diversity that is found between, rather than within sub-populations), allowing the region to be located in a genome. This approach has successfully detected large non-recombining regions in different genera of cichlid fishes from Lake Tanganyika, using pooled samples of at least 25 individuals of each sex and single nucleotide polymorphisms (SNPs) with coverage of at least 10 [76], and identified the SEX locus genome region in another fish, the turbot, whose fully sex-linked region appears to be very small [77].

Population genomic data have recently been published for the guppy [78]. A first analysis inferred a fully Y-linked region by assembling a female reference genome and then searching the sequences of two males and two females for a region of chromosome 12 that had lower sequence coverage in males than females. Based on assuming that the guppy X- and Y-linked sequences are only slightly diverged, and that loss of genes from the Y is minor, the coverage analysis was not intended to detect Y-linkage of regions where genes have been lost by genetic degeneration, but to detect regions where Y-linked sequences are slightly diverged from their X-linked alleles; therefore, identity was required for a sequence to be mapped to the female reference genome (i.e., non-mapping, and consequently low coverage in males, will reflect sequence divergence). This approach identified a 3 Mb candidate Y linked region between 22 and 25 Mb in the female assembly. Divergence was not estimated, and therefore no specific divergence time can yet be inferred. 

The study also identified slightly elevated densities of SNPs in males (a proxy for detecting regions with male-specific SNPs that would suggest diverged fully Y-linked sequences) in a 7 Mb region, from 15 to 22 Mb, adjacent to the region just described; these were suggested to represent candidate younger strata. Together, these candidate strata span nearly half of the chromosome, and it was suggested that they indicate successive sex chromosome recombination suppression events similar to the strata in other organisms reviewed above. Most interestingly, the putative younger strata were detected only in natural guppy populations from up-river sites, while the putative old stratum showed excess SNPs densities in males of all three populations, consistent with the SEX locus being in this region. 

This interpretation is consistent with selection on different SA polymorphic male coloration factors having led to independent evolution of new fully sex-linked strata within different up-river populations where bright coloration is commonest and is less selectively disadvantageous than in the ancestral down-river sites. At least the two up-river populations with the most pronounced SNP density effect (from the Yarra and Aripo Rivers) could thus have independently evolved young strata. However, the previous findings for the guppy outlined above suggested the opposite conclusion, that recombination between coloration factors and the sex-determining locus is rarest in down-river populations. Larger samples, including both sexes, should be studied to confirm the findings just outlined, as the approach rests on detecting linkage disequilibrium between the SNP sequence variants used and the sex-determining locus. Large fully sex-linked regions might be detectable based on such associations in natural populations, because many variants in the region would all suggest sex linkage, contrasting with other genome regions and other chromosomes. However, the approach is problematic for very small samples, because non-zero LD is expected, even for unlinked variants, making it very difficult to localize a small sex-linked region, especially if recombination is infrequent. 

It should be noted that the low-predation populations are thought to have evolved recently after colonisation by guppies from down-river locations [78,79,80]. If recombination is indeed consistently lower in down-river males, the evolutionary change could therefore be the opposite of that assumed: rather than recent evolution of close linkage in down-river fish, it could involve up-river, low predation populations having evolved higher recombination, compared with an ancestral state with less recombination. Even if populations differ in their strengths of sex linkage in the guppy XY pair, the role of SA selection may nevertheless remain unclear.

### 3.3. Could Linkage Disequilibrium with Sexually Antagonistic Polymorphisms Create the Appearance of Sex Linkage?

An alternative interpretation of the findings of Wright et al. [78] is the possibility of strong, but incomplete, LD between the sex-determining locus and molecular variants. If recombination is infrequent, as suggested above, this might be possible [81,82]. If an SA polymorphism is maintained in a partially sex-linked region for enough time, a second peak in molecular variation can form, like that identifying the SEX locus itself, pointing to its location, and the region between the SA locus and the SEX locus may also show elevated values of nucleotide diversity (caused by variants associated with the SEX or maleness factor); the region can therefore potentially be identified by high *F*_ST_ between X- and Y-linked sequences, or, failing that, between the sexes [81,82].

The analyses so far published [78] do not definitively locate the region carrying the sex-determining locus. The coverage differences in the 3 Mb region inferred to be the older stratum are variable, and much smaller than those in other fish, such as the threespine stickleback [37]. This is consistent with the interpretation [78] that the guppy X and Y stopped recombining recently. However, no estimate of the nucleotide divergence between Y- and X-linked sequences in this region are yet available, and the evidence for complete sex linkage is not yet definitive. The evidence for complete sex linkage, and the inference of younger strata occupying 7 Mb chromosome 12 regions, are weaker. The analysis of SNP density that generated this conclusion presumably compared the two captive population females (whose coverage was analysed, see above) with the samples of four males per natural population. The weak associations in the regions identified again leave complete sex linkage unclear. The alternative possibility, that the chromosome’s known SA polymorphisms have led to allele frequency differences between the sexes, is not yet excluded.

Moreover, guppy populations, particularly in low predation sites, could potentially have several different Y haplotypes, associated with different sets of coloration traits. If so, depending on their ages and the extent of differences in neutral variants, diversity values for Y-linked regions could be elevated, which will reduce *F*_ST_ values between the two sexes below the value expected for complete sex linkage, hindering detection of sex linkage. It may be possible to test this possibility by more detailed analyses, including, if recombination is rare enough, determining the phase of variants at different sites in chromosome 12. 

Overall, therefore, the crossover pattern in guppy males suggests that the restrictive conditions for maintenance of SA polymorphisms could be satisfied for most of the guppy XY pair. It does not, however, follow that these polymorphisms have selected for close linkage, which, as already mentioned, may simply reflect an ancestral state in which recombination was rare in males.

It is currently uncertain whether the guppy male sex-determining locus is invariably in the same location. Changes in locations of sex-determining loci have been documented in several fish taxa, including two groups related to guppies, cichlids and Tilapia species [83]. In African cichlids, at least nien autosomes have become sex chromosomes, apparently in the last 15 million years, and large regions (≥19 Mb) with clear footprints of complete XY-linkage were found on different chromosomes in three species [76]. Some fish sex-determining loci could be similar to the apparently moveable housefly M factor (male-determining factor) [84,85,86], but, as mentioned above, there is no evidence that the guppy male-determiner is ever carried on a chromosome other than 12. However, a chromosome 12 location has not yet been verified in many families or populations. 

The observed differences in the locations of male-specific heterochromatin in different guppy material [61] suggest that the SEX locus could be at different chromosome 12 locations in different populations. The different morphologies of the guppy Y and the X [61] also suggest yet another possibility, polymorphic chromosomal inversions, which could restrict recombination in some chromosome 12 regions, depending on their locations and frequencies. Studying associations between molecular variants such as SNPs and sex may allow this to be tested, although chromosome rearrangements could complicate population genomic analyses.

### 3.4. Sex Reversal and the Evolution of Sex Chromosome Sequence Diversity and Divergence

A pattern of crossovers being restricted to the chromosome tips in males has been proposed to explain puzzling observations in some frog populations. Some *Rana temporaria* (common frog) populations have distinct male and female genotypes, with microsatellite alleles at loci across most of the chromosome that carries the sex-determining locus forming a clear male-specific haplotype, implying Y-linkage, but in other populations genetic variants are not associated with the male-determining locus [87,88]. In hylid frogs, occasional sex-reversals create XY females, allowing rare recombination events between the X and Y chromosomes [89]. In *R. temporaria*, crossovers are restricted to the chromosome tips in males, while there is a more uniform distribution in female meiosis [87,90,91], and sex-reversed *R. temporaria* show the recombination pattern of their phenotypic sex, not the sex that their sex chromosomes would indicate, potentially explaining the differences in associations between marker alleles and sex [74]. 

**Figure 4:**
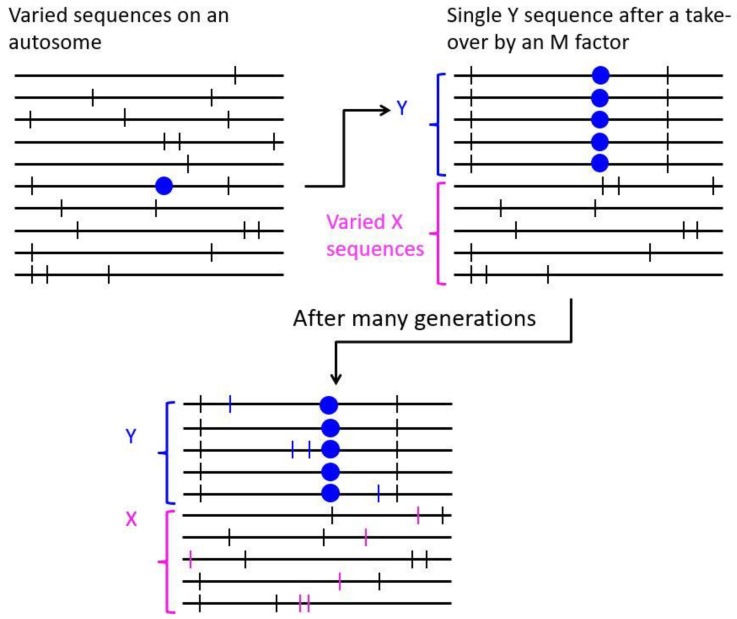
Diagram of predictions about the evolution of sequences in a genome region that is Y linked due to a male-determining factor (blue dot) having arisen in the region. If the male-determining factor spread rapidly, causing a selective sweep, the newly Y linked region would lose most variability at sites with polymorphisms in the population (indicated by black vertical lines), while the polymorphisms would persist in the X-linked region (with the Y alleles generally being the variants commonest among the X chromosomes). New variants that subsequently arise in the non-recombining region will be in linkage disequilibrium (LD) with the male-determining factor. These Y-linked alleles (blue vertical lines) will not be present in the population of X chromosomes and will be detectable as male-specific variants (deleterious mutations might also arise in the Y-linked region, but, for simplicity, are not shown). New variants that arise in the X-linked region are indicated as pink vertical lines. Neither of these two types of new variants can become homozygous unless recombination occurs between the X- and Y-linked regions. If recombination is rare, LD with the male-determining factor will erode slowly but might remain detectable in the region surrounding the with the male-determining factor, allowing it to be located.

Sex reversed XY females are known in guppies suggesting that X–Y recombination can probably sometime occur [50]. If a non-recombining chromosome region (including a centromeric region) acquires a male-determining gene, the whole non-recombining region would immediately show Y-linkage (Figure 4). The turnover event might cause an initial selective sweep, after which new variants will arise in LD with the male-determining factor and will exhibit Y-linkage. “Migration” between the Y- and X-linked populations of sequences through rare crossing over events, and perhaps gene conversion, might then occur, allowing variants to become homozygous in males. Examining sequence data can therefore allow one to test whether recombination occur between such a chromosome pair.

### 3.5. Evolution of Heterochiasmy

Heterochiasmy with all chromosomes failing to cross over in one sex, or in certain genome regions, is known in a diversity of species [92]. It could be a pleiotropic effect of selection to suppress recombination between the sex chromosome pair. Alternatively, a sex-determining factor evolved in a pre-existing non-recombining region (as mentioned above, this is one possibility in the guppy). This alternative has been criticized on the ground that it does not explain the pre-existing sex difference in recombination, or why low recombination is associated with the heterogametic sex. However, if a pre-existing sex difference in recombination has evolved for any reason, including through pleiotropy, an association of low recombination with the heterogametic sex could then be explained by new sex-determining factors subsequently appearing in turnover events. As explained above, the evolution of new sex-linked regions may be widespread in species with pre-existing genome-wide heterochiasmy [74]. 

### 3.6. Male Coloration Factor Genetics and Sex-Limited Expression

This review has already mentioned areas requiring more future work on this fish, and several puzzles remaining to be solved. To conclude, I outline a further unresolved issue. If recombination in guppy males is confined to a limited physical genome, this is difficult to reconcile with the known location of male coloration factors. Coloration factor polymorphisms appear to largely become established in the region proximal to the low recombination region, consistent with the theoretical prediction outlined above that close linkage favours such polymorphisms. The largest recombination frequency between coloration factors and the SEX locus is just over 10% [44], and most are within 6 cM [52]. If recombination is restricted to a physically small region, recombination rates in male guppies will be predicted to be high, as in the mammalian pseudo-autosomal regions (reviewed by [93]). The regions reflected by these genetic distances would then seem unlikely to carry many genes. The entire guppy LG12 has been estimated to carry about 800–900 genes, fairly uniformly distributed across the 26.5 Mb physical assembly [53]. The whole of the 3 Mb recombining region estimated above, based on the cytogenetic observations of guppy males, might therefore include only around 100 genes. As already mentioned, males from natural populations should be examined to find out whether low predation populations have larger recombining regions, perhaps allowing the observed multiple coloration factor polymorphisms [44,49]. Study of this question should involve interesting developmental genetics. 

A related very interesting developmental question for future research is the non-expression of male coloration factors when they are present in females. It is currently unknown whether this sex-limited expression evolved in guppies after coloration traits appeared by mutation, versus the alternative that this fish has an abundant supply of male-limited mutations.

## Figures and Tables

**Table 1:** Results of crosses involving down-river fish from the Aripo river in Trinidad, showing the evidence for a low recombination rate between the sex-determining locus and the partially sex-linked *Sb* factor when the male parents originated from a high-predation population [44]. All parental males carried the *Sb* factor described in the text, and the females’ genotypes for this factor were unknown. The progeny were reared in the laboratory, and the female progeny were treated with testosterone to induce development as males and reveal the presence of the normally male-limited *Sb* factor. The table shows the numbers of male and female progeny exhibiting this trait.

Males		Females		Male parent genotype
*Sb*	Non-*Sb*	*Sb*	Non-*Sb*	
11	0	0	8	X(−)/Y(*Sb*)
7	0	0	6	X(−)/Y(*Sb*)
18	0	0	13	X(−)/Y(*Sb*)
14	0	0	16	X(−)/Y(*Sb*)
16	0	0	18	X(−)/Y(*Sb*)
11	0	0	7	X(−)/Y(*Sb*)
5	0	0	7	X(−)/Y(*Sb*)
9	0	0	5	X(−)/Y(*Sb*)
20	0	0	17	X(−)/Y(*Sb*)
40	0	0	35	X(−)/Y(*Sb*)
32	0	0	29	X(−)/Y(*Sb*)
28	0	0	18	X(−)/Y(*Sb*)
23	0	0	19	X(−)/Y(*Sb*)
7	0	0	14	X(−)/Y(*Sb*)
19	0	0	3	X(−)/Y(*Sb*)
31	0	0	25	X(−)/Y(*Sb*)
15	0	1	14	X(−)/Y(*Sb*)
11	0	0	13	X(−)/Y(*Sb*)
20	0	0	16	X(−)/Y(*Sb*)
30	0	0	19	X(−)/Y(*Sb*)
27	0	0	23	X(−)/Y(*Sb*)
20	0	0	10	X(−)/Y(*Sb*)
23	0	0	20	X(−)/Y(*Sb*)
11	0	0	9	X(−)/Y(*Sb*)
18	0	0	7	X(−)/Y(*Sb*)
12	0	0	12	X(−)/Y(*Sb*)
20	0	0	13	X(−)/Y(*Sb*)
21	0	0	10	X(−)/Y(*Sb*)
7	0	0	15	X(−)/Y(*Sb*)
8	0	0	5	X(−)/Y(*Sb*)
6	0	0	14	X(−)/Y(*Sb*)
14	0	0	6	X(−)/Y(*Sb*)
11	0	0	12	X(−)/Y(*Sb*)
565	0	1	458	1024

Total numbers are shown in the bottom rows of each set. In the column at the right showing the male parent genotypes inferred from the progeny, blue font indicates progeny in which the *Sb* factor behaves as Y-linked in the male parent of a family, and red indicates X-linkage, while X(−) and Y(−) symbols in black indicate, respectively, X and Y chromosomes with no *Sb* factor. Numbers in red font indicate recombinant progeny.

**Table 2:** Results of crosses and treatments of female progeny in the same manner as for the results shown in Table 1, except that the parents were up-river fish, showing the evidence for a genetic difference in the recombination rate between the sex-determining locus and the partially sex-linked *Sb* factor in the up- and down-river populations [44]. The colours and symbols are explained in the legend of Table 1.

Males		Females		Male parent genotype
*Sb*	Non-*Sb*	*Sb*	Non-*Sb*	
6	0	1	10	X(−)/Y(*Sb*)
8	0	0	15	X(−)/Y(*Sb*)
12	0	0	20	X(−)/Y(*Sb*)
14	0	0	2	X(−)/Y(*Sb*)
13	0	0	14	X(−)/Y(*Sb*)
11	0	0	3	X(−)/Y(*Sb*)
12	0	0	6	X(−)/Y(*Sb*)
7	0	0	8	X(−)/Y(*Sb*)
0	15	4	0	X(*Sb*)/Y(−)
3	11	10	0	X(*Sb*)/Y(−)
1	10	14	0	X(*Sb*)/Y(−)
0	10	7	0	X(*Sb*)/Y(−)
17	0	21	0	X(*Sb*)/Y(*Sb*)
7	0	8	0	X(*Sb*)/Y(*Sb*)
9	0	17	0	X(*Sb*)/Y(*Sb*)
5	0	3	0	X(*Sb*)/Y(*Sb*)
6	0	5	0	X(*Sb*)/Y(*Sb*)
10	0	5	0	X(*Sb*)/Y(*Sb*)
14	0	13	0	X(*Sb*)/Y(*Sb*)
155	46	108	78	387
